# Increased hypoxic proliferative response and gene expression in erythroid progenitor cells of Andean highlanders with chronic mountain sickness

**DOI:** 10.1152/ajpregu.00250.2019

**Published:** 2019-10-16

**Authors:** Daniela Bermudez, Priti Azad, Rómulo Figueroa-Mujíca, Gustavo Vizcardo-Galindo, Noemí Corante, Cristina Guerra-Giraldez, Gabriel G. Haddad, Francisco C. Villafuerte

**Affiliations:** ^1^Laboratorio de Fisiología Comparada, Facultad de Ciencias y Filosofía, Universidad Peruana Cayetano Heredia, Lima, Peru; ^2^Division of Respiratory Medicine, Department of Pediatrics, University of California, San Diego, La Jolla, California; ^3^Laboratorio de Inflamación Cerebral, Facultad de Ciencias y Filosofía, Universidad Peruana Cayetano Heredia, Lima, Peru; ^4^Department of Neurosciences, University of California San Diego, La Jolla, California; ^5^Rady Children's Hospital, San Diego, La Jolla, California; ^6^Instituto de Investigaciones de la Altura (IIA), Universidad Peruana Cayetano Heredia, Lima, Peru

**Keywords:** Andean, chronic mountain sickness, erythropoiesis, excessive erythrocytosis, high altitude

## Abstract

Excessive erythrocytosis (EE) is the main sign of chronic mountain sickness (CMS), a maladaptive clinical syndrome prevalent in Andean and other high-altitude populations worldwide. The pathophysiological mechanism of EE is still controversial, as physiological variability of systemic respiratory, cardiovascular, and hormonal responses to chronic hypoxemia complicates the identification of underlying causes. Induced pluripotent stem cells derived from CMS highlanders showed increased expression of genes relevant to the regulation of erythropoiesis, angiogenesis, cardiovascular, and steroid-hormone function that appear to explain the exaggerated erythropoietic response. However, the cellular response to hypoxia in native CMS cells is yet unknown. This study had three related aims: to determine the hypoxic proliferation of native erythroid progenitor burst-forming unit-erythroid (BFU-E) cells derived from CMS and non-CMS peripheral blood mononuclear cells; to examine their sentrin-specific protease 1 (SENP1), GATA-binding factor 1 (GATA1), erythropoietin (EPO), and EPO receptor (EPOR) expression; and to investigate the functional upstream role of SENP1 in native progenitor differentiation into erythroid precursors. Native CMS BFU-E colonies showed increased proliferation under hypoxic conditions compared with non-CMS cells, together with an upregulated expression of SENP1, GATA1, EPOR; and no difference in EPO expression. Knock-down of the SENP1 gene abolished the augmented proliferative response. Thus, we demonstrate that native CMS progenitor cells produce a larger proportion of erythroid precursors under hypoxia and that SENP1 is essential for proliferation. Our findings suggest a significant intrinsic component for developing EE in CMS highlanders at the cellular and gene expression level that could be further enhanced by systemic factors such as alterations in respiratory control, or differential hormonal patterns.

## INTRODUCTION

The excessive production of red blood cells (RBCs) or excessive erythrocytosis (EE) is considered an indicator of poor adaptation to life at high altitude (6, 35, 37, 43, 45). EE is the main sign of a clinical syndrome known as chronic mountain sickness (CMS) or Monge’s disease, common to Andean and other high-altitude populations around the world (13, 36, 44, 57). Hypoxemia and neurological symptoms such as headache, fatigue, and alterations of sleep and memory usually accompany EE (3, 26, 30, 33, 49, 53). Pulmonary hypertension (38, 40) and cerebral, cardiovascular, and pulmonary accidents caused by blood hyperviscosity and thromboembolic events (2, 25, 26, 2) are also frequent in CMS patients. The prevalence of CMS increases with altitude, and the condition develops insidiously from early adulthood, progressing with age (27, 34, 36). Symptoms can significantly affect the quality of life in native highlanders, as the condition frequently becomes incapacitating. Several studies have shown that the common practice of hemodilution at the altitude of residence alleviates CMS-related symptoms (11, 24, 54, 56), indicating that symptomatology is secondary to EE. The pathophysiological mechanism that results in the occurrence of EE, however, is still controversial, although systemic physiological alterations in terms of respiratory, cardiovascular, and hormonal responses to chronic hypoxemia have been proposed as potential causes to explain the exacerbated erythropoietic response (4, 18, 29, 47). These alterations might be associated with differential polymorphisms in genes related to the regulation of erythropoiesis, angiogenesis, cardiovascular, and steroid-hormone function, among others, identified by whole-genome studies in CMS and non-CMS highlanders (4). In particular, a number of studies suggest that sentrin-specific protease 1 (SENP1), which regulates the activity of transcription factors such as hypoxia-inducible factor (HIF) and GATA-binding factor (GATA), has a central role in the excessive production of RBCs, possibly by modulating different stages of erythropoiesis, including steps of the erythropoietin (EPO) signaling pathway and apoptosis in erythroid progenitors (5, 8, 58).

EPO is an essential hypoxia-responsive erythropoietic hormone that mediates the survival, proliferation, and differentiation of erythroid progenitors (17, 23). Under hypoxic conditions, EPO levels increase, stimulating the production of RBCs and elevating hematocrit to a new steady state (32). However, among Andean highlanders, most studies show that despite severe hypoxemia, serum EPO concentrations in CMS and non-CMS individuals are similar, although the former show significantly higher hematocrit values. Elevated serum EPO accompanies only extreme hematocrit values (12, 28). We have previously shown that decreased plasma concentration of the soluble EPO receptor (sEPOR), an endogenous EPO antagonist, is among the factors that might explain EE despite altitude-normal serum EPO in CMS highlanders, because increased blood EPO availability to bind membrane EPOR would result in a stronger erythropoietic stimulus (50, 51). Another suggested mechanism includes increased local expression of EPO in the bone marrow, associated with increased hypoxia inducible factor α levels (48). However, erythroid progenitor cells of CMS individuals could, hypothetically, be more sensitive to EPO, and hence their proliferative response to hypoxia would be stronger than in non-CMS cells.

Under in vitro hypoxic conditions, CMS erythroid progenitors derived from human induced pluripotent stem cells (iPSCs) obtained from skin fibroblasts show increased proliferation and increased SENP1 expression, associated with stabilization and upregulation of GATA-binding factor 1 (GATA1) and GATA1-responsive genes such as the mitochondrial antiapoptotic factor Bcl-xL (5). These findings suggest a crucial role for SENP1 in erythroid proliferation seen in CMS. Still, whether physiological responses of iPSCs-derived CMS erythroid progenitors are the same as in native CMS cells needs confirmation. Besides, since the expression of Bcl-xL requires the activation of EPOR, also a GATA1 target (16, 58, 59), increased EPO sensitivity in erythroid progenitors is possibly associated with upregulated EPOR expression. Therefore, the aim of the present study was to determine the hypoxic proliferative response of native erythroid progenitors (burst-forming units-erythroid [BFU-E]) derived from CMS and non-CMS peripheral blood mononuclear cells (PBMCs); to examine the expression of SENP1, GATA1, EPOR, and EPO in BFU-E cell cultures; and also to investigate the functional upstream role of SENP1 in native erythroid progenitor differentiation into erythroid precursors in the absence of the influence of systemic factors.

## MATERIALS AND METHODS

### 

#### Ethical approval.

The study was approved by the Institutional Ethics Committee of Universidad Peruana Cayetano Heredia and by the University of California San Diego, Human Research Protection Program. All participants signed an informed consent form in Spanish.

#### Study participants.

Thirty-six participants, 17 CMS and 19 non-CMS native highlanders, were included in the study. A minimum sample size of 16 participants per group for BFU-E colony proliferation rate and total colony area as primary outcome variables was estimated using a Cohen's *d* effect size value of 1 with a statistical power of 80% at *P* < 0.05. All participants were men, high-altitude natives, and lifelong residents of Cerro de Pasco, Peru (4,380 m), 20 to 65 yr old, and had at least two previous known generations of high-altitude (>3,000 m) Andean ancestry. Participants were excluded if they had history of pulmonary, cardiovascular, or renal disease; were current smokers; were miners; had undergone blood transfusions or phlebotomies in the previous six months; had traveled to lower altitudes (<3,000 m) for more than seven days during the previous six months; or had demonstrated abnormal cardiac or pulmonary function during screening procedures.

#### Preliminary screening, hematocrit, and CMS score.

Clinical examination was performed during a preliminary screening session to rule out prior history of cardiovascular or pulmonary disease. During this session, pulse oxygen saturation ($\text{S}{\text{p}}_{{\text{O}}_{2}}$), heart rate, and systolic and diastolic blood pressure were measured. Hematocrit was determined from duplicate micro-centrifuged blood samples obtained from a fingertip capillary blood draw. Participants with hematocrit ≥ 63% (Hb concentration ≥ 21g/dl) were classified as individuals with EE (26). General health and CMS score questionnaires were also applied. CMS score determines the absence or presence and severity of the syndrome and is based on the occurrence of EE, as well as the following signs and symptoms: headache, shortness of breath or palpitations, sleep disturbances, paresthesia, cyanosis, dilated veins, and tinnitus (26). All participants were also interviewed about their history of high-altitude residence and ancestral background (self-identified ancestry and geographical location of their parents and grandparents).

#### Samples.

Blood samples for PBMC isolation were obtained in three 10-ml sodium heparin-coated tubes. Two additional 6-ml blood samples were obtained in clot-activator-coated tubes to determine EPO and iron profile. Samples were taken between 5 AM and 7 AM to avoid variation in serum EPO due to circadian rhythm. Serum was separated by centrifugation and stored in liquid N_2_ until analysis.

#### Serum EPO and iron homeostasis indexes.

A specific sandwich ELISA kit was used for serum EPO determination as described by the manufacturer (DRG International, Springfield Township, NJ). Each sample was run in duplicate. Iron, ferritin, and transferrin were measured in serum samples (Medlab clinical laboratories, ISO 9001:2000, Lima, Peru).

#### PBMCs isolation and cell culture.

PBMCs were isolated using Histopaque 1077 (Sigma-Aldrich, St. Louis, MO) by gradient centrifugation. PBMCs were then cultured in Methocult H-4534 medium (StemCell Technologies, Vancouver, Canada) at a final density of 2.5 × 10^5^ cells/ml in the presence of 3 U/ml of rhEPO (StemCell Technologies) for 14 days. Cell cultures were maintained at 37°C with 5% CO_2_ and 10% O_2_ for normoxic conditions in a Biospherix X3 hood (Biospherix, Parish, NY). Cellular normoxia for PBMCs was calculated as the fractional equivalent of the mean between average arterial and venous Po_2_ ($Pa_{O}{}_{{}_{2}}$ = 95 mmHg and ${\text{Pv}}_{{\text{O}}_{2}}$ = 45 mmHg, respectively) near sea level (barometric pressure [BP] = 747 mmHg), taking into account water vapor pressure (P_v_H_2_O) at 37°C. Therefore: [[($Pa_{O}{}_{{}_{2}}$ + ${\text{Pv}}_{{\text{O}}_{2}}$)/2]/(BP − P_v_H_2_O)] = 0.099 or 10%. For incubation under hypoxic conditions, O_2_ levels were lowered down to 1%.

#### BFU-E colony identification and count.

Colony identification and count were performed at ×20 magnification on *days 7* and *10* using an inverted LS 560 microscope (Etaluma, Carlsbad, CA) inside the hypoxic hood. On *day 14*, cultures were removed from the hood and analyzed using an AxioZoom Stereomicroscope (Carl Zeiss Microscopy, Jena, Germany) at ×10 magnification. Digital images were analyzed with Zeiss ZEN software to calculate the area occupied by each colony. The rate of change in the number of colonies, or colony proliferation, was calculated as the slope of the relationship between number of colonies and days of incubation (from *day 7* to *day 14*). No colonies were present at *day 0*. Total colony size was calculated as the product of the number of individual colonies by their area on *day 14*.

#### Real-time PCR.

Expression of EPO and EPOR mRNA was determined in BFU-E colony cells by reverse transcription quantitative PCR (RT-qPCR), using 18S rRNA as the reference gene. Total RNA was isolated using TRIzol Reagent (Invitrogen, Carlsbad, CA) and an RNeasy Mini kit (Qiagen, Hilden, Germany). cDNA was generated using 500 µg of total RNA using Multiscribe reverse transcriptase (Applied Biosystems, Foster City, CA). Quantitative PCR was performed in 10-µl reactions using LightCycler 480 SYBR Green I Master (Roche Life Science, Indianapolis, IN) and 100 pM of primers directed to EPO, EPOR mRNA, and 18S rRNA. PCR reactions were run in triplicate for each sample using 40 cycles of 2 min at 95°C and 1 min at 60°C on the Pikoreal 96 platform (Thermo Fisher Scientific, Waltham, MA). SENP1 and GATA1 cDNA was produced from total RNA through reverse transcription-PCR using a Superscript Vilo IV system (Invitrogen). Quantitative PCR was performed using a GeneAmp 7900 Sequence detection system using POWER SYBR Green chemistry (Applied Biosystems). Results were calculated as fold change and also as relative gene expression. Fold change was calculated to compare the expression levels of the target genes between the CMS and non-CMS groups using the Livak method (2^−ΔΔCt^) taking the average of Δ threshold cycle (Ct) values of the non-CMS group at 10% O_2_ as control group, 2^−(ΔCt CMS – ΔCt non-CMS)^. The relative expression ratio was used to compare the expression levels between each one of the target genes and the housekeeping gene, 2ΔCt  target2ΔCt  reference (41). Considering that the Ct of the reference gene is the same for both the CMS and non-CMS groups at both O_2_ concentrations, then the ΔCt equals 0 and the former equation can be simplified to 2^ΔCt target^ (38).

#### Generation of erythroid precursor (CD235a cells) from native PBMC-derived erythroid progenitor (CD34+) cells of CMS and non-CMS subjects under normoxia and hypoxia.

Erythroid precursors (CD3235a cells) were generated from PBMCs by gradient centrifugation using Histopaque. Dynabeads CD34+ Isolation Kit (Invitrogen, CA) was used to purify the CD34^+^ fraction. CD34^+^ cells were expanded for a week (*days 0–7*) in Stem span medium containing Hydrocortisone (10^−3^M), SCF (50 ng/ml), FLT3L (50 ng/ml), IL3 (10 ng/ml), BMP4 (1 ng/ml), IL-11 (40 ng/ml), and EPO (2 U/ml). After expansion, cells were further differentiated using the protocol from Giarratana et al. (17a). Cells were then cultured in erythroid differentiation medium (EDM) on the basis of IMDM supplemented with stabilized glutamin, 330 μg/mL holo-human transferrin, 10 μg/ml recombinant human insulin, 2 IU/ml heparin, and 5% plasma. In the second step (*days 7* to *11*), cells were resuspended at 10^5^/ml in EDM supplemented with SCF and EPO. In the third step (*days 11* to *18*), cells are cultured in EDM supplemented with EPO alone. Cell counts were adjusted to 7.5 × 10^5^ to 1 × 10^6^ and 5~10 × 10^6^ cells/ml on *days 11* and *15*, respectively. Beyond *day 18*, the culture medium containing EPO was renewed twice a week and cultures were maintained at 37°C in 5% CO_2_ under normoxic or hypoxic conditions; results are presented in terms of the actual rate of expansion after plating. For flow cytometric analysis, erythroid bodies were dissociated using Accuatase Cell Dissociation reagent (Invitrogen, Carlsbad, CA), washed with PBS supplemented with 2% FBS, and filtered through a 70-µm cell strainer (Falcon; BD). Cells were treated with propidium iodide (Sigma-Aldrich) before analysis. Cells were hCD235a-PE (glycophorin A) from BD and analyzed by a FACSCanto cell analyzer (BD) using FACSDiva software (version 6.0; BD).

#### SENP1 knock-down.

For SENP1 knock-down, packaging and lentivirus generation was done by Salk Institute Gene Transfer, Targeting, and Therapeutics Core. Transduced cells were selected at 0.5 µg/ml puromycin (Sigma-Aldrich). The cells were transduced at CD34^+^ cell stage and selected by puromycin selection before expansion as discussed in the protocol above.

#### Statistical analysis.

STATA 15 software was employed for statistical analysis. Normality of distribution and homogeneity of variance were assessed for comparison between continuous variables. Student’s *t* test for equal variances was applied as parametric test and Wilcoxon *t* test as nonparametric test to evaluate differences between CMS and non-CMS cell culture outcomes and to compare expression levels of EPO and EPOR in CMS and non-CMS cells cultured under hypoxia and normoxia, whereas Student’s *t*-test was performed to compare the expression of SENP1 and GATA1. In general, values of *P* < 0.05 were considered statistically significant.

## RESULTS

Table 1 presents general characteristics of all participants. CMS highlanders showed higher hematocrit, higher CMS score, and lower $\text{S}{\text{p}}_{{\text{O}}_{2}}$ compared with the non-CMS group. Also, CMS participants were younger on average, had similar body mass index, and showed similar mean serum EPO concentration. Iron homeostasis parameters were also similar between groups.

**Table 1.** Characteristics of study participants

	Non-CMS (*n* = 19)	CMS (*n* = 17)
Age, yr	51.9 ± 2.2	44.8 ± 2.7^*^
BMI, kg/m^2^	26.1 ± 0.7	26.9 ± 0.8
Hematocrit, %	54.7 ± 0.6	68.1 ± 0.8**
CMS score	0.37 ± 0.1	7.1 ± 0.8*
$\text{S}{\text{p}}_{{\text{O}}_{2}}$, %	87.4 ± 0.8	83.8 ± 0.6^***^
HR, beats/min	65.6 ± 2.6	71.6 ± 2.1
Serum EPO, pg/dl	12.3 ± 2.9	21.6 ± 6.7
SBP, mmHg	110.6 ± 3.4	110.7 ± 1.9
DBP, mmHg	72.4 ± 3.6	72.5 ± 2.1
Serum iron, µg/dl	100.7 ± 10.9	120.3 ± 15.7
Serum ferritin, ng/ml	162.9 ± 24.2	124.6 ± 19.3
Serum transferrin saturation, %	26.4 ± 2.7	29.6 ± 3.8

Values are expressed as means ± SE; *n* = number of participants. BMI, body mass index; CMS, chronic mountain sickness; DBP, diastolic blood pressure; EPO, erythropoietin; HR, heart rate; SBP, systolic blood pressure; $\text{S}{\text{p}}_{{\text{O}}_{2}}$, pulse O_2_ saturation.

* *P* < 0.05;

** *P* < 0.001;

*** *P* < 0.01.

**Fig. 1.**
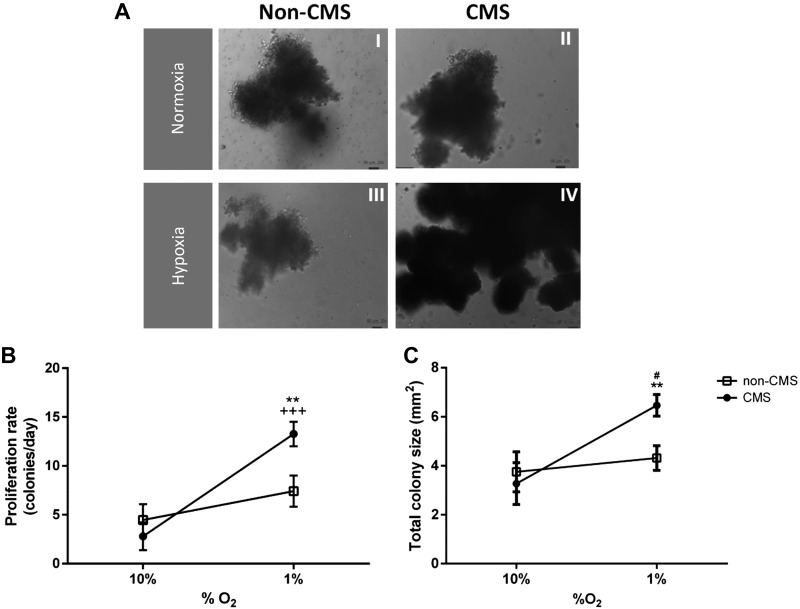
Erythroid progenitor culture under hypoxia and cellular normoxia. *A*: non-chronic mountain sickness (CMS) (*i*) and CMS (*ii*) burst-forming unit-erythroid (BFU-E) colonies cultured under normoxia. Non-CMS (*iii*) and CMS (*iv*) colonies cultured under hypoxic conditions. Images were acquired at *day 14* and ×20 magnification. *B*: proliferation rate of BFU-E cultured colonies from *day 7* to *day 14* under hypoxia and cellular normoxia. **P < 0.01 CMS (*n* = 17) vs. non-CMS (*n* = 19) in hypoxia, +++*P* < 0.001 hypoxia vs. normoxia in CMS cells. *C*: total BFU-E colony size under cellular normoxia and hypoxia at *day 14* of cell culture. **P < 0.01 CMS versus non-CMS in hypoxia. #*P* < 0.01 normoxia vs. hypoxia in non-CMS cells.

Figure 1*A* shows representative images of CMS and non-CMS BFU-E colonies cultured under normoxia and hypoxia. When cultured under hypoxic conditions (1% O_2_), CMS-derived BFU-E colonies showed a greater proliferation rate compared with non-CMS colonies (367.3 ± 89.1 vs. 100.9 ± 19.1%, *P* < 0.01; Fig. 1*B*). Under cellular normoxia (10% O_2_) no difference was observed between groups. CMS colonies showed a greater proliferation rate when cultured in hypoxia compared with normoxia, whereas non-CMS colonies showed similar proliferation in both conditions. Total colony size was larger in CMS cells cultured in hypoxia compared with non-CMS cells (6.4 ± 0.5 vs. 4.2 ± 0.5 mm^2^, *P* < 0.01), and also larger than CMS-derived colonies cultured in normoxia (6.4 ± 0.5 vs. 3.2 ± 0.8 mm^2^, *P* < 0.01; Fig. 1*C*).

**Fig. 2.**
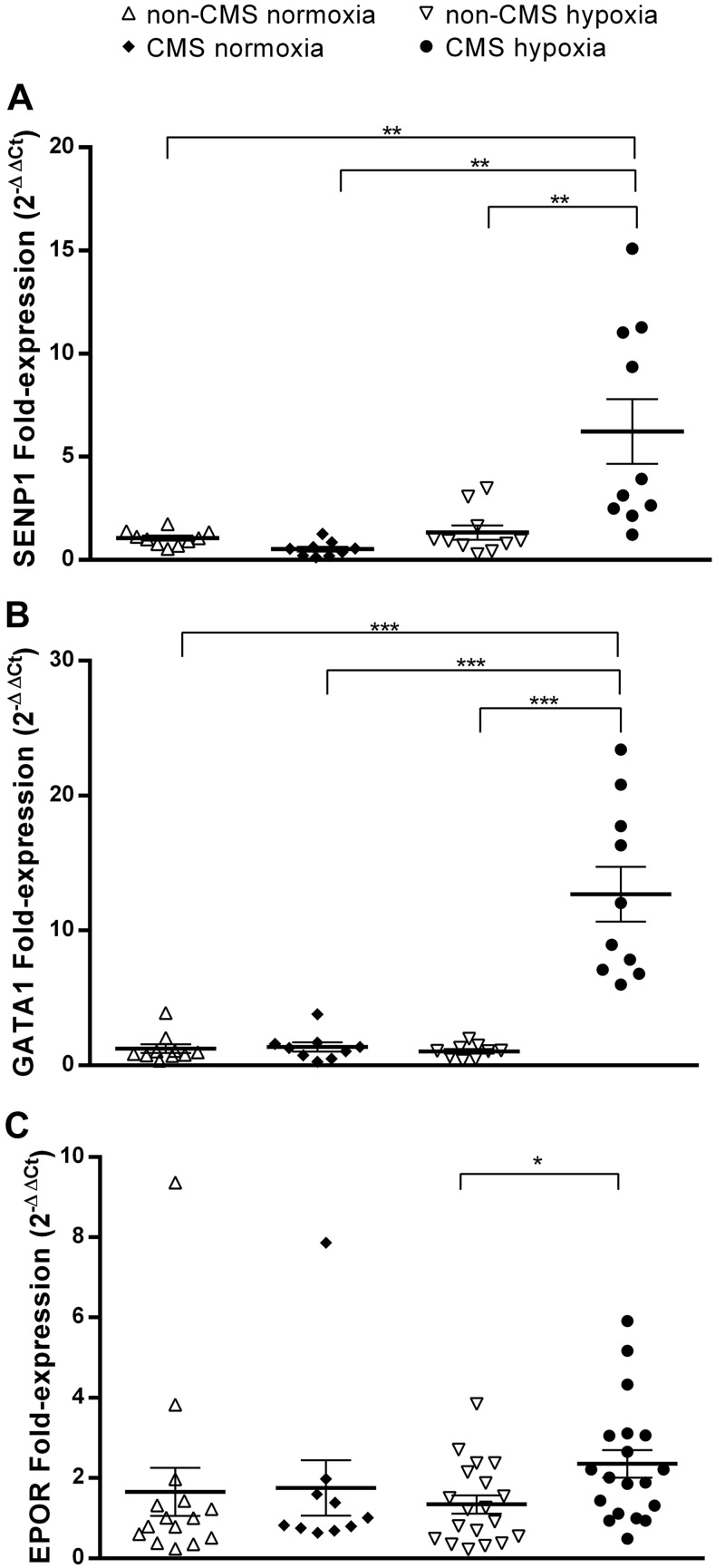
Gene fold-expression in burst-forming unit-erythroid (BFU-E) colonies under hypoxia and cellular normoxia after 14 days of cell culture. Comparison of sentrin-specific protease 1 (SENP1) (*A*), GATA-binding factor 1 (GATA1) (*B*), and erythropoietin (EPO) receptor (EPOR) (*C*) fold expression between chronic mountain sickness (CMS) and non-CMS cells in hypoxia and cellular normoxia. SENP1 expression in hypoxia in CMS and non-CMS samples, *n* = 10. SENP1 expression under normoxia in CMS and non-CMS samples, *n* = 9 and *n* = 10, respectively. GATA1 expression under hypoxia in CMS and non-CMS samples, *n* = 10 and *n* = 9, respectively. GATA1 expression under normoxia in CMS and non-CMS samples, *n* = 9 and *n* = 10, respectively. EPOR expression in hypoxia in CMS and non-CMS samples, *n* = 17 and *n* = 19, respectively. EPOR expression in normoxia in CMS and non-CMS samples, *n* = 12 and *n* = 15, respectively. Values expressed as means ± SE. *P < 0.05, **P < 0.01, ***P < 0.001.

Our RT-qPCR results showed that fold change expression of SENP1 mRNA in CMS cells in hypoxia is significantly higher than in CMS cells under normoxic conditions (6.23- vs. 0.52-fold, *P* < 0.01; Fig. 2*A*). When compared with non-CMS cells in hypoxia and cellular normoxia, the hypoxic expression of SENP1 in CMS cells was also significantly higher (6.23- vs. 1.32-fold, 6.23- vs. 1.05-fold, *P* < 0.01, respectively). GATA1 mRNA hypoxic expression in CMS cells is higher compared with non-CMS cells (12.69- vs. 1.04-fold, *P* < 0.001; Fig. 2*B*), and also significantly higher compared with both CMS and non-CMS cells in normoxia (12.69- vs. 1.36-fold, and 12.69- vs. 1.24-fold, *P* < 0.001, respectively). Under hypoxic conditions, CMS cells showed higher EPOR mRNA expression compared with non-CMS cells (2.4-fold, *P* < 0.05; Fig. 2*C*). Non-CMS cells showed no difference in SENP1, GATA1, or EPOR expression under hypoxic or normoxic conditions. Similar results were obtained for the relative expression of the same genes. EPO expression showed no difference between groups under hypoxia or normoxia, either as fold or relative expression.

**Fig. 3.**
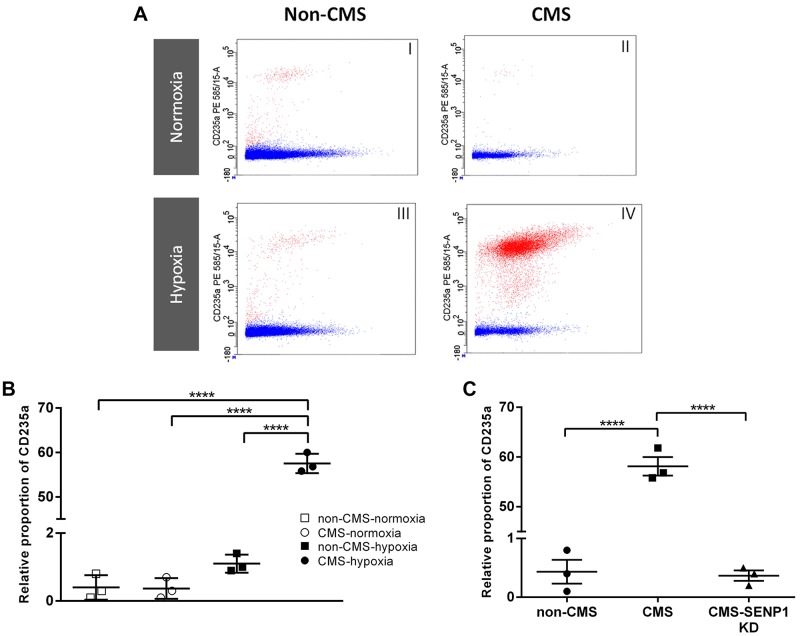
FACS analysis of native cells and the effect of sentrin-specific protease 1 (SENP1) knockdown. *A*: representative images of FACS analysis on relative proportion of live CD235a (red dots) calculated after normalizing with propidium iodide staining (blue dots). *B*: relative proportion of chronic mountain sickness (CMS) and non-CMS CD235a cells in normoxia and hypoxia (*n* = 3). *C*: SENP1 knock-down resulted in a significant decrease of CD235a CMS cells relative proportion down to non-CMS levels (*n* = 3). Values expressed as means ± SE; ****P < 0.0001.

Figure 3*A* shows representative FACS analysis images of native erythroid cells. FACS analysis showed that in normoxia, CMS and non-CMS CD34+ cells generate similar relative proportions of erythroid precursors (CD235a cells, Fig. 3*A**, i* and *ii*, and Fig. 3*B*). However, under hypoxic conditions, CMS CD34+ cells generated a significantly larger proportion of CD235a cells (Fig. 3*A**, iii and iv*, and Fig. 3*B*). Figure 3*C* shows that SENP1 knock-down in CMS cells reduced the relative proportion of CD235a to similar levels found in non-CMS cells.

## DISCUSSION

The excessive hematological response observed in a significant part of the Andean population who suffer from EE and CMS is undoubtedly an indicator of maladaptation to life at high altitude. We generated native erythroid progenitor cells from CMS and non-CMS individuals to search for core cellular mechanisms in the absence of systemic factors. Our main finding shows an intrinsic exacerbated erythropoietic response to hypoxia in erythroid progenitor CMS cells together with increased gene expression of SENP1, GATA1, and EPOR, with SENP1 as a crucial upstream regulator.

The role of serum EPO has been controversial concerning the development of EE in Andeans. Typically, serum EPO values are similar in CMS and non-CMS highlanders, and there is no correlation with hematocrit or hemoglobin concentration (18, 22, 28, 48, 51). Only in few cases, CMS individuals show elevated EPO, which usually associates with extreme hematocrits (12, 28, 51), and therefore it is unlikely that this sole factor explains the excessive RBC production. Recently, we have shown that plasma sEPOR concentration and the EPO-to-sEPOR ratio, as an index of EPO availability, are better systemic predictors of hematocrit in CMS than EPO alone (50). Here, we confirm similar serum EPO values in CMS and non-CMS highlanders, and their lack of association with EE.

We also demonstrate that under similar EPO concentration in vitro, CMS cells show a stronger proliferative response, reflected in the higher proliferation rate and larger total colony area during culture under hypoxic conditions, compared with non-CMS cells. Interestingly, the latter did not show a significant increase in either proliferation rate or total colony size, which suggests a relatively blunted erythropoietic response. A similar but more pronounced blunting was reported in hypoxic PBMCs-derived BFU-E colonies obtained from Tibetan natives, typically known for lower hematocrits compared with Andeans at similar altitudes (31). These cells showed decreased EPO sensitivity and significantly less proliferation and colony size compared with colonies from control lowlanders. The study showed that this reduced erythropoietic response was related to missense variants of the *EGLN1* gene. These variants are associated with the relatively low Hb values observed in Tibetans and are considered protective alleles against high-altitude polycythemia (46). However, we have recently shown that these *EGLN1* variants are absent or in very low frequency in Andeans of this same population of Cerro de Pasco (21), and therefore the partially blunted erythropoietic response seen in Andean non-CMS BFU-E cells is most possibly related to different alleles or epigenetic modifications (10, 14, 15).

A key point in our study is the increased expression of SENP1 and GATA1 in CMS erythroid progenitors, confirming the findings on iPSC-derived CMS erythroid cells. Under hypoxic conditions at 5% O_2_, iPSC-derived CMS erythroid cells display a robust proliferative response to form RBCs (5). Also, the study showed that SENP1 plays a critical role in the differential erythropoietic response of CMS versus non-CMS cells. The results strongly suggest that GATA1 is a downstream SENP1 target and has a critical antiapoptotic effect in these cells. GATA1 drives the expression of many erythropoietic and antiapoptotic genes, and it is also required for EPOR expression (9, 60). However, the study did not find a differential expression of EPOR between CMS and non-CMS cells. In our study, we observed a modest increase in EPOR fold- and relative expression in CMS compared with non-CMS cells under hypoxic conditions. This apparent discrepancy can be due to the erythroid progenitor differentiation stage and the severity of hypoxic culture conditions. The iPSC-derived CMS erythroid cells used were at the erythroid body stage II, which corresponds roughly to the proerythroblast to reticulocyte stage. The expression of EPOR increases from very low levels at the megakaryocyte-erythroid progenitor cell stage (1, 20), reaching a peak during the maturation from colony-forming units-erythroid (CFU-E) to proerythroblasts, and starts its decline during the progression into the remaining stages of the erythroid lineage (7, 23). Thus, less EPOR expression is expected at both the proerythroblast and reticulocyte stage, compared with progenitors at earlier midstages of erythroid differentiation (BFU-Es and CFU-Es). The more severe hypoxic culture conditions used in the present study (1% vs. 5% O_2_) might also represent a stronger stimulus for EPOR expression. In line with the study on iPSCs, we did not find any expression difference in the *EPO* gene. Increased EPO expression has only been reported in mononuclear cells obtained from bone marrow biopsies of Han-Chinese CMS patients (48). Mononuclear cells require EPO for both commitment into the hematopoietic lineage and primary differentiation (19, 52, 55). Moreover, EPO is required to trigger EPOR expression in the later stages of erythroid differentiation (39, 42). Therefore, at this early stage, increased EPO expression and a paracrine EPO effect are required for differentiation, and if these events are more pronounced in CMS highlanders, they would contribute to the excessive RBC production. However, at BFU-E stage, less EPO is required to maintain intracellular EPO signaling and progenitor differentiation. Therefore, it makes sense that augmented erythroid proliferation of CMS BFU-E cells under hypoxic conditions relate to increased expression of upstream regulators such as SENP1, and to transcription factors such as GATA1 for upregulation of EPOR and other antiapoptotic factors. In the present study, we show that isolated native CMS erythroid progenitors (CD34+ cells) produce a significantly larger relative proportion of late-stage erythroid precursors (CD235a cells) under hypoxic conditions, and that SENP1 knock-down brings differentiation and proliferation of erythroid precursors down to non-CMS levels. This observation confirms the key functional upstream role played by SENP1 in the augmented erythropoietic activity observed in native CMS cells.

### Perspectives and Significance

Native CMS BFU-E erythroid progenitors show increased proliferative response under hypoxic conditions compared with non-CMS cells, and upregulated expression of SENP1, GATA1, and EPOR. Also, isolated native CMS CD34+ cells produce a more substantial proportion of erythroid precursors under hypoxia, and SENP1 knock-down abolishes this augmented proliferation, eliminating the cellular CMS phenotype. These findings suggest a significant intrinsic component at the cellular and gene expression level for the development of EE in CMS highlanders. Thus, our results are consistent with the possibility that this cellular EE phenotype might represent the basis for excessive RBC production, which could be further enhanced by poor systemic oxygenation during daytime or sleep as a consequence of altered respiratory control, or by differential hormonal patterns. The similarities between the hypoxic response of native CMS erythroid cells and the iPSCs-derived CMS erythroid lineage in terms of proliferation, differentiation, and gene expression validate the use of the latter to obtain further insight on the fundamental pathophysiological mechanism of EE. They also provide an unlimited resource to expand the search for novel cellular pathways and pharmacological targets with potential clinical applications on highlanders with this condition.

## GRANTS

This work was funded by a Wellcome Trust Grant 107544/Z/15/Z to F. C. Villafuerte and by National Heart, Lung, and Blood Institute Grant 1R01HL146530-01 to GGH.

## DISCLOSURES

The authors declare that the research was conducted in the absence of any commercial or financial relationships that could be construed as a potential conflict of interest.

## AUTHOR CONTRIBUTIONS

F.C.V. conceived and designed research; D.B., P.A., R.F.-M., G.V.-G., C.G.-G., and F.C.V. performed experiments; D.B., P.A., N.C., and F.C.V. analyzed data; D.B., P.A., N.C., and F.C.V. interpreted results of experiments; D.B. prepared figures; D.B., C.G.-G., and F.C.V. drafted manuscript; D.B., P.A., R.F.-M., G.V.-G., N.C., C.G.-G., G.G.H., and F.C.V. edited and revised manuscript; D.B., P.A., R.F.-M., G.V.-G., N.C., C.G.-G., G.G.H., and F.C.V. approved final version of manuscript.

## References

[1] Akashi K, Traver D, Miyamoto T, Weissman IL A clonogenic common myeloid progenitor that gives rise to all myeloid lineages. Nature 404: 193–197, 2000. doi:10.1038/35004599. 10724173

[2] Arias-Stella J Chronic Mountain Sickness: pathology and definition. In; High Alltitude Physiology: Cardiac and respiratory Aspects, edited by Porter R, Knight J Edinburgh, London: Churchill Livingstone, 1971, p. 31–40.

[3] Arregui A, León-Velarde F, Cabrera J, Paredes S, Vizcarra D, Umeres H Migraine, polycythemia and chronic mountain sickness. Cephalalgia 14: 339–341, 1994. doi:10.1046/j.1468-2982.1994.1405339.x. 7828191

[4] Azad P, Stobdan T, Zhou D, Hartley I, Akbari A, Bafna V, Haddad GG High-altitude adaptation in humans: from genomics to integrative physiology. J Mol Med (Berl) 95: 1269–1282, 2017. doi:10.1007/s00109-017-1584-7. 28951950 PMC8936998

[5] Azad P, Zhao HW, Cabrales PJ, Ronen R, Zhou D, Poulsen O, Appenzeller O, Hsiao YH, Bafna V, Haddad GG Senp1 drives hypoxia-induced polycythemia via GATA1 and Bcl-xL in subjects with Monge’s disease. J Exp Med 213: 2729–2744, 2016. doi:10.1084/jem.20151920. 27821551 PMC5110013

[6] Beall CM, Cavalleri GL, Libin D, Elston RC, Gao Y, Knight J, Chaohua L, Li JC, Liang Y, McCormack M, Montgomery HE, Pan H, Robbins PA, Shianna KV, Tam SC, Tsering N, Veeramah KR, Wang W, Wangdui P, Weale ME, Xu Y, Xu Z, Yang L, Zaman MJ, Zeng C, Zhang L, Zhang X, Zhaxi P, Zheng YT Natural selection on EPAS1 (HIF2alpha) associated with low hemoglobin concentration in Tibetan highlanders. Proc Natl Acad Sci USA 107: 11459–11464, 2010. doi:10.1073/pnas.1002443107. 20534544 PMC2895075

[7] Broudy VC, Lin N, Brice M, Nakamoto B, Papayannopoulou T Erythropoietin receptor characteristics on primary human erythroid cells. Blood 77: 2583–2590, 1991. doi:10.1182/blood.V77.12.2583.2583. 1646044

[8] Cheng J, Kang X, Zhang S, Yeh ET SUMO-specific protease 1 is essential for stabilization of hypoxia-inducible factor-1α during hypoxia. Cell 131: 584–595, 2007. doi:10.1016/j.cell.2007.08.045. 17981124 PMC2128732

[9] Chiba T, Nagata Y, Kishi A, Sakamaki K, Miyajima A, Yamamoto M, Engel JD, Todokoro K Induction of erythroid-specific gene expression in lymphoid cells. Proc Natl Acad Sci USA 90: 11593–11597, 1993. doi:10.1073/pnas.90.24.11593. 8265595 PMC48030

[10] Crawford JE, Amaru R, Song J, Julian CG, Racimo F, Cheng JY, Guo X, Yao J, Ambale-Venkatesh B, Lima JA, Rotter JI, Stehlik J, Moore LG, Prchal JT, Nielsen R Natural selection on genes related to cardiovascular health in high-altitude adapted Andeans. Am J Hum Genet 101: 752–767, 2017. doi:10.1016/j.ajhg.2017.09.023. 29100088 PMC5673686

[11] Cruz JC, Diaz C, Marticorena E, Hilario V Phlebotomy improves pulmonary gas exchange in chronic mountain polycythemia. Respiration 38: 305–313, 1979. doi:10.1159/000194097. 538338

[12] Dainiak N, Spielvogel H, Sorba S, Cudkowicz L Erythropoietin and the polycythemia of high-altitude dwellers. Adv Exp Med Biol 271: 17–21, 1989. doi:10.1007/978-1-4613-0623-8_3. 2486283

[13] De Ferrari A, Miranda JJ, Gilman RH, Dávila-Román VG, León-Velarde F, Rivera-Ch M, Huicho L, Bernabé-Ortiz A, Wise RA, Checkley W Prevalence, clinical profile, iron status, and subject-specific traits for excessive erythrocytosis in andean adults living permanently at 3,825 meters above sea level. Chest 146: 1327–1336, 2014. doi:10.1378/chest.14-0298. 24874587 PMC4219344

[14] Eichstaedt CA, Antão T, Pagani L, Cardona A, Kivisild T, Mormina M The Andean adaptive toolkit to counteract high altitude maladaptation: genome-wide and phenotypic analysis of the Collas. PLoS One 9: e93314, 2014. doi:10.1371/journal.pone.0093314. 24686296 PMC3970967

[15] Eichstaedt CA, Pagani L, Antao T, Inchley CE, Cardona A, Mörseburg A, Clemente FJ, Sluckin TJ, Metspalu E, Mitt M, Mägi R, Hudjashov G, Metspalu M, Mormina M, Jacobs GS, Kivisild T Evidence of early-stage selection on EPAS1 and GPR126 genes in Andean high altitude populations. Sci Rep 7: 13042, 2017. doi:10.1038/s41598-017-13382-4. 29026132 PMC5638799

[16] Ferreira R, Ohneda K, Yamamoto M, Philipsen S GATA1 function, a paradigm for transcription factors in hematopoiesis. Mol Cell Biol 25: 1215–1227, 2005. doi:10.1128/MCB.25.4.1215-1227.2005. 15684376 PMC548021

[17] Ghaffari S, Huang LJS, Zhang J, Lodish HF Erythropoietin receptor signaling processes. In: Erythropoietins and Erythropoiesis, edited by Molineux G, Foote MA, Elliott SG Basel: Birkhauser, 2003.

[17a] Giarratana MC, Rouard H, Dumont A, Kiger L, Safeukui I, Le Pennec PY, François S, Trugnan G, Peyrard T, Marie T, Jolly S, Hebert N, Mazurier C, Mario N, Harmand L, Lapillonne H, Devaux JY, Douay L Proof of principle for transfusion of in vitro-generated red blood cells. Blood 118: 5071–5079, 2011. doi:10.1182/blood-2011-06-362038. 21885599 PMC3217398

[18] Gonzales GF, Gasco M, Tapia V, Gonzales-Castañeda C High serum testosterone levels are associated with excessive erythrocytosis of chronic mountain sickness in men. Am J Physiol Endocrinol Metab 296: E1319–E1325, 2009. doi:10.1152/ajpendo.90940.2008. 19318512 PMC2692401

[19] Grover A, Mancini E, Moore S, Mead AJ, Atkinson D, Rasmussen KD, O’Carroll D, Jacobsen SE, Nerlov C Erythropoietin guides multipotent hematopoietic progenitor cells toward an erythroid fate. J Exp Med 211: 181–188, 2014. doi:10.1084/jem.20131189. 24493804 PMC3920567

[20] Heberlein C, Fischer KD, Stoffel M, Nowock J, Ford A, Tessmer U, Stocking C The gene for erythropoietin receptor is expressed in multipotential hematopoietic and embryonal stem cells: evidence for differentiation stage-specific regulation. Mol Cell Biol 12: 1815–1826, 1992. doi:10.1128/MCB.12.4.1815. 1312671 PMC369625

[21] Heinrich EC, Wu L, Lawrence ES, Cole AM, Anza-Ramires C, Villafuerte FC, Simonson TS Genetic variants at the EGLN1 locus associated with high-altitude adaptation in Tibetans are absent or found at low frequency in highland Andeans. Ann Hum Genet 83: 171–176, 2019. doi:10.1111/ahg.12299. 30719713 PMC7920394

[22] Hsieh MM, Callacondo D, Rojas-Camayo J, Quesada-Olarte J, Wang X, Uchida N, Maric I, Remaley AT, Leon-Velarde F, Villafuerte FC, Tisdale JF SENP1, but not fetal hemoglobin, differentiates Andean highlanders with chronic mountain sickness from healthy individuals among Andean highlanders. Exp Hematol 44: 483–490.e2, 2016. doi:10.1016/j.exphem.2016.02.010. 26952840 PMC6471513

[23] Jelkmann W Regulation of erythropoietin production. J Physiol 589: 1251–1258, 2011. doi:10.1113/jphysiol.2010.195057. 21078592 PMC3082088

[24] Klein HG Isovolemic hemodilution in high altitude polycythemia. In: Proceedings of the International Symposium on Acclimatization, Adaptation and Tolerance to High Altitude. Washington, DC: US Department of Health and Human Services, 1983, p. 47–51.

[25] Leon-Velarde F, Arregui A. Desadaptacion a la vida en las grandes alturas. In: Travaux de l’Institut Francaise d’Etudes Andines, edited by Leon-Velarde F and Arregui A Lima, Peru: Institut Francais d’etudes Andines (IFEA), 1994.

[26] León-Velarde F, Maggiorini M, Reeves JT, Aldashev A, Asmus I, Bernardi L, Ge RL, Hackett P, Kobayashi T, Moore LG, Penaloza D, Richalet JP, Roach R, Wu T, Vargas E, Zubieta-Castillo G, Zubieta-Calleja G Consensus statement on chronic and subacute high altitude diseases. High Alt Med Biol 6: 147–157, 2005. doi:10.1089/ham.2005.6.147. 16060849

[27] León-Velarde F, Monge C, Ruiz y Ruiz H Aging at high altitudes and the risk of chronic mountain sickness. J Wilderness Med 4: 183–188, 1993. doi:10.1580/0953-9859-4.2.183.

[28] León-Velarde F, Monge CC, Vidal A, Carcagno M, Criscuolo M, Bozzini CE Serum immunoreactive erythropoietin in high altitude natives with and without excessive erythrocytosis. Exp Hematol 19: 257–260, 1991. 2055289

[29] León-Velarde F, Richalet JP Respiratory control in residents at high altitude: physiology and pathophysiology. High Alt Med Biol 7: 125–137, 2006. doi:10.1089/ham.2006.7.125. 16764526

[30] León-Velarde F, Rivera-Ch M, Huicho L, Villafuerte FC Chronic mountain sickness. In: High Altitude Human Adaptation to Hypoxia, edited by Swenson E, Bartsch P New York: Springer, 2014, p. 429.

[31] Lorenzo FR, Huff C, Myllymäki M, Olenchock B, Swierczek S, Tashi T, Gordeuk V, Wuren T, Ri-Li G, McClain DA, Khan TM, Koul PA, Guchhait P, Salama ME, Xing J, Semenza GL, Liberzon E, Wilson A, Simonson TS, Jorde LB, Kaelin WG Jr, Koivunen P, Prchal JT A genetic mechanism for Tibetan high-altitude adaptation. Nat Genet 46: 951–956, 2014. doi:10.1038/ng.3067. 25129147 PMC4473257

[32] Milledge J, Bartsch P. Blood and haemostasis. In: High Altitude: Human Adaptation to Hypoxia, edited by Swenson ER, Bärtsch P New York: Springer, 2014, p. 203–216.

[33] Monge C Chronic mountain sickness. Physiol Rev 23: 166–184, 1943. doi:10.1152/physrev.1943.23.2.166.

[34] Monge-C C, Arregui A, León-Velarde F Pathophysiology and epidemiology of chronic mountain sickness. Int J Sports Med 13, Suppl 1: S79–S81, 1992. doi:10.1055/s-2007-1024603. 1483802

[35] Monge C, León-Velarde F Physiological adaptation to high altitude: oxygen transport in mammals and birds. Physiol Rev 71: 1135–1172, 1991. doi:10.1152/physrev.1991.71.4.1135. 1924550

[36] Monge C, León-Velarde F, Arregui A Increasing prevalence of excessive erythrocytosis with age among healthy high-altitude miners. N Engl J Med 321: 1271, 1989. doi:10.1056/NEJM198911023211817. 2797095

[37] Moore LG Measuring high-altitude adaptation. J Appl Physiol (1985) 123: 1371–1385, 2017. doi:10.1152/japplphysiol.00321.2017. 28860167 PMC5792094

[38] Naeije R, Vanderpool R Pulmonary hypertension and chronic mountain sickness. High Alt Med Biol 14: 117–125, 2013. doi:10.1089/ham.2012.1124. 23795731

[39] Noguchi CT, Wang L, Rogers HM, Teng R, Jia Y Survival and proliferative roles of erythropoietin beyond the erythroid lineage. Expert Rev Mol Med 10: e36, 2008. doi:10.1017/S1462399408000860. 19040789 PMC3065109

[40] Penaloza D, Arias-Stella J The heart and pulmonary circulation at high altitudes: healthy highlanders and chronic mountain sickness. Circulation 115: 1132–1146, 2007. doi:10.1161/CIRCULATIONAHA.106.624544. 17339571

[41] Pfaffl MW A new mathematical model for relative quantification in real-time RT-PCR. Nucleic Acids Res 29: e45, 2001. doi:10.1093/nar/29.9.e45. 11328886 PMC55695

[42] Richmond TD, Chohan M, Barber DL Turning cells red: signal transduction mediated by erythropoietin. Trends Cell Biol 15: 146–155, 2005. doi:10.1016/j.tcb.2005.01.007. 15752978

[43] Ronen R, Zhou D, Bafna V, Haddad GG The genetic basis of chronic mountain sickness. Physiology (Bethesda) 29: 403–412, 2014. doi:10.1152/physiol.00008.2014. 25362634 PMC4280153

[44] Sahota IS, Panwar NS Prevalence of Chronic Mountain Sickness in high altitude districts of Himachal Pradesh. Indian J Occup Environ Med 17: 94–100, 2013. doi:10.4103/0019-5278.130839. 24872667 PMC4035612

[45] Simonson TS Altitude adaptation: a glimpse through various lenses. High Alt Med Biol 16: 125–137, 2015. doi:10.1089/ham.2015.0033. 26070057 PMC4490743

[46] Simonson TS, Yang Y, Huff CD, Yun H, Qin G, Witherspoon DJ, Bai Z, Lorenzo FR, Xing J, Jorde LB, Prchal JT, Ge R Genetic evidence for high-altitude adaptation in Tibet. Science 329: 72–75, 2010. doi:10.1126/science.1189406. 20466884

[47] Stobdan T, Akbari A, Azad P, Zhou D, Poulsen O, Appenzeller O, Gonzales GF, Telenti A, Wong EHM, Saini S, Kirkness EF, Venter JC, Bafna V, Haddad GG New insights into the genetic basis of Monge’s disease and adaptation to high-altitude. Mol Biol Evol 34: 3154–3168, 2017. doi:10.1093/molbev/msx239. 29029226 PMC5850797

[48] Su J, Li Z, Cui S, Ji L, Geng H, Chai K, Ma X, Bai Z, Yang Y, Wuren T, Ge RL, Rondina MT The local HIF-2α/EPO pathway in the bone marrow is associated with excessive erythrocytosis and the increase in bone marrow microvessel density in chronic mountain sickness. High Alt Med Biol 16: 318–330, 2015. doi:10.1089/ham.2015.0015. 26625252 PMC4855733

[49] Villafuerte FC, Corante N Chronic mountain sickness: clinical aspects, etiology, management, and treatment. High Alt Med Biol 17: 61–69, 2016. doi:10.1089/ham.2016.0031. 27218284 PMC4913504

[50] Villafuerte FC, Corante N, Anza-Ramírez C, Figueroa-Mujíca R, Vizcardo-Galindo G, Mercado A, Macarlupú JL, León-Velarde F Plasma soluble erythropoietin receptor is decreased during sleep in Andean highlanders with chronic mountain sickness. J Appl Physiol (1985) 121: 53–58, 2016. doi:10.1152/japplphysiol.00107.2016. 27125843 PMC4967249

[51] Villafuerte FC, Macarlupú JL, Anza-Ramírez C, Corrales-Melgar D, Vizcardo-Galindo G, Corante N, León-Velarde F Decreased plasma soluble erythropoietin receptor in high-altitude excessive erythrocytosis and chronic mountain sickness. J Appl Physiol (1985) 117: 1356–1362, 2014. doi:10.1152/japplphysiol.00619.2014. 25324511 PMC4254844

[52] Ward D, Carter D, Homer M, Marucci L, Gampel A Mathematical modeling reveals differential effects of erythropoietin on proliferation and lineage commitment of human hematopoietic progenitors in early erythroid culture. Haematologica 101: 286–296, 2016. doi:10.3324/haematol.2015.133637. 26589912 PMC4815720

[53] Winslow R, Monge CC Hypoxia, Polycythemia, and Chronic Mountain Sickness. Baltimore, MD: Johns Hopkins University Press, 1987.

[54] Winslow RM, Monge CC, Brown EG, Klein HG, Sarnquist F, Winslow NJ, McKneally SS Effects of hemodilution on O_2_ transport in high-altitude polycythemia. J Appl Physiol (1985) 59: 1495–1502, 1985. doi:10.1152/jappl.1985.59.5.1495. 4066580

[55] Wu H, Liu X, Jaenisch R, Lodish HF Generation of committed erythroid BFU-E and CFU-E progenitors does not require erythropoietin or the erythropoietin receptor. Cell 83: 59–67, 1995. doi:10.1016/0092-8674(95)90234-1. 7553874

[56] Wu TY Excessive polycythemia of high altitude: an analysis of 82 cases [in Chinese]. Zhonghua Xue Ye Xue Za Zhi 3: 27–32, 1979.

[57] Wu TY, Li W, Li Y, Ge R-L, Cheng Q, Wang S, Zhao G, Wei L, Jin Y, Don G Epidemiology of chronic mountain sickness: ten years' study in Qinghai-Tibet. In: Progress in Mountain Medicine and High Altitude Physiology, edited by Ohno H, Kobayashi T, Masuyama S, Nakashima M. Matsumoto, Japan: Press Committee of the 3rd World Congress on Mountain Medicine and High Altitude Physiology, 1998, p. 120–125.

[58] Yu L, Ji W, Zhang H, Renda MJ, He Y, Lin S, Cheng EC, Chen H, Krause DS, Min W SENP1-mediated GATA1 deSUMOylation is critical for definitive erythropoiesis. J Exp Med 207: 1183–1195, 2010. doi:10.1084/jem.20092215. 20457756 PMC2882842

[59] Zhao C, Li Z, Ji L, Ma J, Ge RL, Cui S PI3K-Akt signal transduction molecules maybe involved in downregulation of erythroblasts apoptosis and perifosine increased its apoptosis in chronic mountain sickness. Med Sci Monit 23: 5637–5649, 2017. doi:10.12659/MSM.905739. 29176544 PMC5713146

[60] Zon LI, Youssoufian H, Mather C, Lodish HF, Orkin SH Activation of the erythropoietin receptor promoter by transcription factor GATA-1. Proc Natl Acad Sci USA 88: 10638–10641, 1991. doi:10.1073/pnas.88.23.10638. 1660143 PMC52985

